# Surface Diffusion Directed Growth of Anisotropic Graphene Domains on Different Copper Lattices

**DOI:** 10.1038/srep21136

**Published:** 2016-02-17

**Authors:** Da Hee Jung, Cheong Kang, Ji Eun Nam, Heekyung Jeong, Jin Seok Lee

**Affiliations:** 1 Department of Chemistry, Sookmyung Women’s University, Seoul 140-742, Republic of Korea

## Abstract

Anisotropic graphene domains are of significant interest since the electronic properties of pristine graphene strongly depend on its size, shape, and edge structures. In this work, considering that the growth of graphene domains is governable by the dynamics of the graphene-substrate interface during growth, we investigated the shape and defects of graphene domains grown on copper lattices with different indices by chemical vapor deposition of methane at either low pressure or atmospheric pressure. Computational modeling identified that the crystallographic orientation of copper strongly influences the shape of the graphene at low pressure, yet does not play a critical role at atmospheric pressure. Moreover, the defects that have been previously observed in the center of four-lobed graphene domains grown under low pressure conditions were demonstrated for the first time to be caused by a lattice mismatch between graphene and the copper substrate.

As the electronic properties of pristine graphene are strongly dependent on its size, shape, and edge structures^1^, variously shaped graphene domains with defined edge configurations have attracted considerable attention^2^,^3^,^4^. They are expected to provide a pathway toward greater insight into the electronic properties of graphene and, hence, toward device performance optimization^5^,^6^,^7^. The edge geometry significantly affects the *p*-electron structure at the edge^1^,^8^; the zigzag edge in a semi-infinite graphene sheet leads to a localized state and the armchair edge, on the other hand, shows no such localized state^8^,^9^. Consequently, graphene domains with edges of various geometries can be expected to exhibit unique reactivity, since they display unique physical characteristics such as particular electronic structures and magnetic properties^5^. With the consideration that the reactivity of graphene is governable by edges of various geometries, there have been many efforts to grow variously shaped graphene domains and define their edge structures. Of the various graphene synthesis techniques, Cu-assisted chemical vapor deposition (CVD) is the most reasonable and appropriate method to produce large-scale and low-defect graphene films^10^,^11^, since it is highly reproducible and yields high-quality films of controllable thickness and domain shapes^12^,^13^. Hexagonal graphene domains, which are usually synthesized using a CVD method, are believed to exhibit predominantly zigzag edge symmetry^4^,^14^. But, graphene domains with anisotropic geometries, such as rectangular^15^ and two-lobed curvilinear structures^16^, are expected to exhibit both zigzag and armchair edge structures because the edges exhibit the same configuration if the angles between them are 2*n* × 30° (*n* = 1, 2, 3, …)^4^,^14^.

Most CVD synthesis of graphene to date has been conducted using methane at either low pressure (LPCVD) or atmospheric pressure (APCVD), with these two conditions reported to produce very different results in terms of the domain shape and the growth mechanism. For instance, graphene domains grown under LPCVD conditions typically has a dendritic four- or six-lobed structure, whereas under APCVD conditions hexagonal domains with six-fold symmetry are dominantly formed, with their edges macroscopically oriented parallel to the zigzag directions^17^. However, what effect the copper substrate has on graphene growth under different pressures remains unclear, especially with regards to the surface diffusion of carbon adatoms on different Cu lattices.

In this study, we investigated a correlation between the surface diffusion of carbon adatoms and the growth of anisotropic graphene domains synthesized using a CVD method. We varied the reaction pressure in CVD synthesis, which in turn caused variations in the growth mechanism and kinetics, and found that the crystallographic orientation of a copper substrate differently influences the shape and defects of graphene domains formed on it under LPCVD and APCVD conditions. We also calculated the energy barrier for surface diffusion of carbon adatoms on different Cu lattices, and propose a surface diffusion directed growth of anisotropic graphene domains on different Cu lattices with experimental observations.

## Results

**Figure 1:**
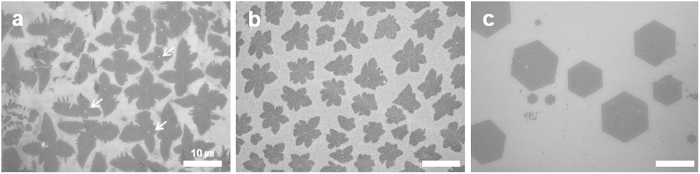
Variation in the shape of graphene domains grown using low pressure and atmospheric pressure chemical vapor deposition. SEM images of (**a**) four-lobed and (**b**) six-lobed graphene domains grown under LPCVD, and (**c**) hexagonal graphene domains grown under APCVD on Cu substrate. Scale bars represent 10 μm.

We synthesised graphene domains on Cu foil by CVD method under low and atmospheric pressures, using methane as the carbon precursor gas^11^,^17^. In the SEM images of the graphene domains grown under LPCVD condition (Fig. 1a,b), dendritic edges can be clearly observed on the surface as four- or six-lobed structures. Moreover, even though the dominance of each structure varies between particular areas, the relative density of each is comparable. However, the four-lobed graphene tends to be slightly larger in size and also quite interestingly contains small holes in its central area, as indicated by the white arrows in Fig. 1a (See the Supplementary Figure S1 for the size distributions). In contrast, Fig. 1c shows that the graphene domains grown under APCVD condition are predominantly a hexagonal structure with a mean domain size of more than 10 μm, as defined by the longest distance between two opposite vertices.

**Figure 2:**
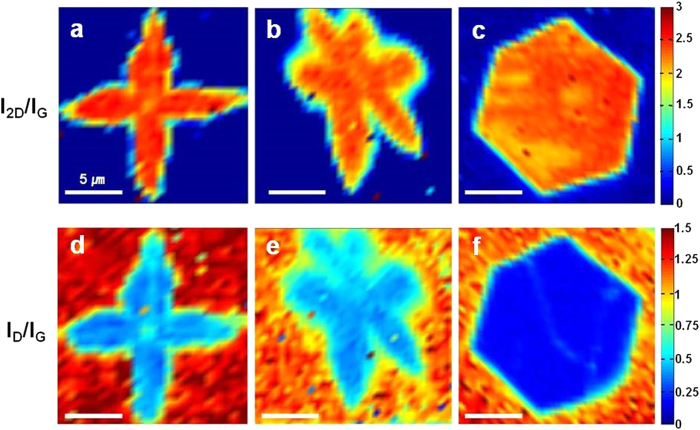
Raman maps of (**a–c**) 2D/G intensity ratio and (**d–f**) D/G intensity ratio for (**a,d**) four-lobed, (**b,e**) six-lobed, and (**c,f**) hexagonal graphene domains, respectively. Scale bars represent 5 μm.

Micro Raman maps were used to further identify the uniformity of the graphene domains produced. Typically, the presence of defects is indicated by the presence of an additional D (~1350 cm^−1^) peak, and thus the ratio of the 2D (~2680 cm^−1^) to G (~1580 cm^−1^) peak intensity (I_2D_/I_G_) and D to G peak intensity (I_D_/I_G_) was determined for each of the graphene domains produced. Their spatial dependencies (Raman maps) are given in Fig. 2, in which the different colors indicate different intensities. The Raman maps in Fig. 2(a–c) confirm that all of the graphene domains are single-layer, as their I_2D_/I_G_ values are greater than 2.0. And, Fig. 2(d–f) show the mapping images used to determine I_D_/I_G_, in which the higher value under LPCVD (~0.4) than APCVD (~0.2) indicates a higher defects density in the former. This was attributed to the greater likelihood of Cu sublimation under low pressure, with recent reports suggesting that copper at 1000 °C in a vacuum has an evaporation rate approaching as high as 4 μm/h, while at higher pressures, sublimation of copper is suppressed^18^,^19^ (See the Supplementary Figure S2 for the proof of severe evaporation of copper at low pressure). This evaporation of Cu also has the potential to promote desorption of carbon species adsorbed on top of Cu substrate^20^, and may therefore cause an increase in the defects density on as-grown graphene domains. The effects of Cu evaporation depending on the background pressure on the surface roughness of Cu substrate which might affect subsequently the defects density on the graphene domains were further investigated by atomic force microscopy (AFM) (See the Supplementary Figure S3). The defects density was also found to increase in the center of the four-lobed graphene domains (Fig. 2d), as evidenced by an I_D_/I_G_ of ~0.7, with additional Raman maps of this region provided in the Supplementary Information (Figure S4 for the four-lobed graphene and Figure S5 for the six-lobed graphene). However, the I_D_/I_G_ value in our case is higher than that of normal CVD graphene (~0.05). This is due to the fact that partial graphene growths have more Raman active edge^21^, which contributes to the higher defects density, besides higher Cu evaporation under LPCVD^19^,^20^. The I_D_/I_G_ value of general graphene film in our system is in the Supplementary Information (See the Supplementary Information Figure S6).

**Figure 3:**
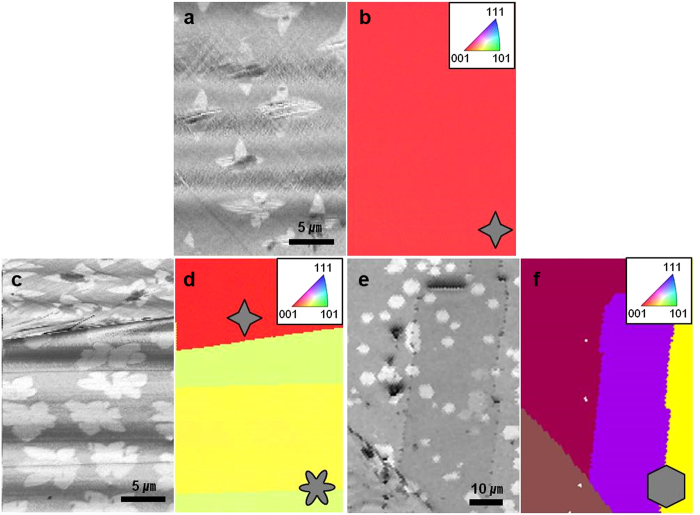
Cu lattice dependence of graphene domains grown on polycrystalline Cu substrate under (**a–d**) LPCVD and (**e,f**) APCVD conditions. SEM images of (**a**) four-lobed, (**c**) six-lobed, and (**e**) hexagonal graphene domains grown on different Cu lattices, and (**b,d,f**) their respective surface normal projected EBSD maps (inverse pole figure). A standard EBSD color key is used, as shown in inset.

In order to better understand the fact that the dominance of four- or six-lobed structures varies between particular areas on Cu substrate, electron backscatter diffraction (EBSD) images were obtained and are shown in Fig. 3 along with their respective SEM images. In Fig. 3(a,b), it is clear that the four-lobed graphene domains are grown on Cu grain orientated to the Cu (001) plane (as marked in red). Meanwhile, the Cu grains marked in green and yellow that are associated with six-lobed graphene domains represent the Cu (102) plane, as shown in Fig. 3d. These domains were found to only grow on these specific Cu planes, whereas the hexagonal domains grown under atmospheric pressure grew regardless of the Cu lattice orientation (Fig. 3e,f). It is therefore suggested that although the Cu lattice plays an important role in determining the shape of graphene domains under low pressure, it has little influence over growth under atmospheric pressure.

**Figure 4:**
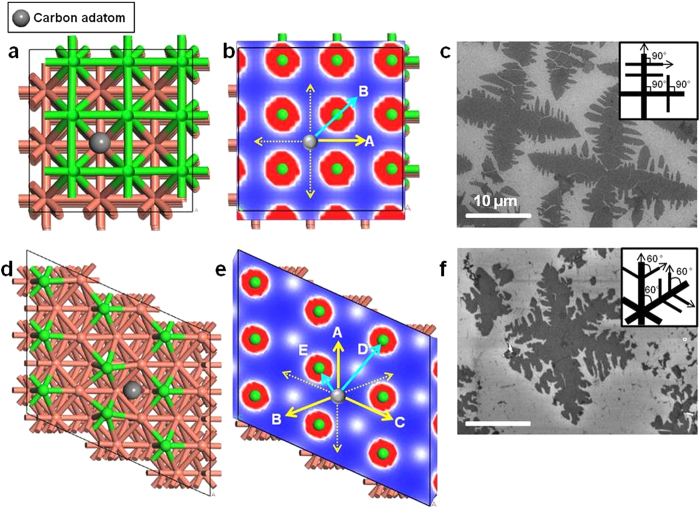
Computational modeling for the diffusion of carbon adatom on the Cu (001) and Cu (102) lattice. Three-dimensional side-on view of the crystal structure of (**a**) Cu (001) and (**d**) Cu (102) lattice. Green circles indicate Cu atoms protruding from surface, and gray circle represents carbon adatom adsorbed on Cu lattices. Total electron density plot of (**b**) Cu (001) and (**e**) Cu (102) lattice. Green circles surrounded by red circles correspond to the surface Cu atoms (region with high electron density), while blue area is region with depleted electron density. The dotted and solid yellow arrows are the preferential diffusion directions of carbon adatoms with a low energy barrier, while the cyan arrows are the poor diffusion directions with a high energy barrier. SEM images of dendritic (**c**) four-lobed and (**f**) six-lobed graphene domains. (Insets) Schematic illustration of fractal growth of graphene domains at low pressure. Scale bars represent 10 μm.

Computational modeling for the diffusion of carbon adatom on the Cu (001) and Cu (102) lattice orientations was used to further explore how the Cu lattice influences the shape of the four- and six-lobed graphene domains, respectively, at low pressure (Fig. 4). In Fig. 4b, we can see that a single carbon adatom on a Cu (001) surface can potentially diffuse in one of two ways. These are indicated by A and B, which differ in terms of their respective energy barriers of 1.89 and 2.92 eV, respectively. Surface diffusion of carbon adatom toward A direction is therefore clearly preferred over B direction, with the carbon adatom capable of diffusing along four possible directions with same energy barrier. (Indicated by solid and dotted yellow arrows) In the case of a Cu (102) surface, a carbon adatom has five possible ways to diffuse (designated A–E). The energy barriers toward the A (1.64 eV), B (1.66 eV) and C (1.70 eV) directions are all quite amenable to diffusion; however, the need to pass over a Cu atom protruding from surface, as marked in green, significantly increases the energy barrier in the D (4.21 eV) and E (5.76 eV) directions because of strong electrostatic repulsion. Given this, there are in fact a total six ways in which carbon can diffuse with a relatively low energy barrier. (Indicated by solid and dotted yellow arrows) We also included investigation on Cu (111), which is most often studied facet in supplementary information (See the Supplementary Information Figure S7).

This change in the influence of the crystallographic orientation of Cu substrate on the anisotropic growth of graphene domains with reaction pressure is due to a variation in the pressure of carbon precursor gas, which provides an explanation as to why the Cu substrate does not always have the same effect. For example, in the case of LPCVD, carbon adatoms diffuse only along those directions with a low energy barrier, which these being determined by the crystallographic orientation of Cu substrate (Sites indicated by yellow arrows in Fig. 4b,e). Consequently, the graphene domains take on symmetrical patterns with four- or six-lobes that reflect the underlying Cu substrate; while the dendritic shape is presumed to be due to the anisotropy of surface diffusion on a Cu surface^22^ (Fig. 4c,f). These anisotropic surface diffusion also influenced the secondary lobed pattern in graphene domains. Conversely, in general APCVD, an excess of carbon supersaturates the Cu surface and minimizes the influence that its crystallographic orientation has on energy barriers of carbon adatom for surface diffusion although various shaped graphene domains such as rectangle or square can also form occasionally by changing reaction parameters^15^,^23^. That is, there are sufficient carbon adatoms to overcome the high energy diffusion barriers. This ultimately results in a hexagonal shape, which is determined by the intrinsic six-fold symmetry in the atomic structure of the graphene rather than the underlying Cu substrate. Thus, the compact hexagonal shapes formed at atmospheric pressure in general indicate that graphene growth is not surface-diffusion limited under APCVD^24^. Under the surface-diffusion limited conditions with a low pressure of carbon precursor gas, the carbon adatom adsorbed onto graphene domain does not relax sufficiently and migrate to find an energetically more favorable location along the edges or corners of domains before additional adatoms adsorb toward graphene domain by surface diffusion, resulting in dendritic structures^25^,^26^.

**Figure 5:**
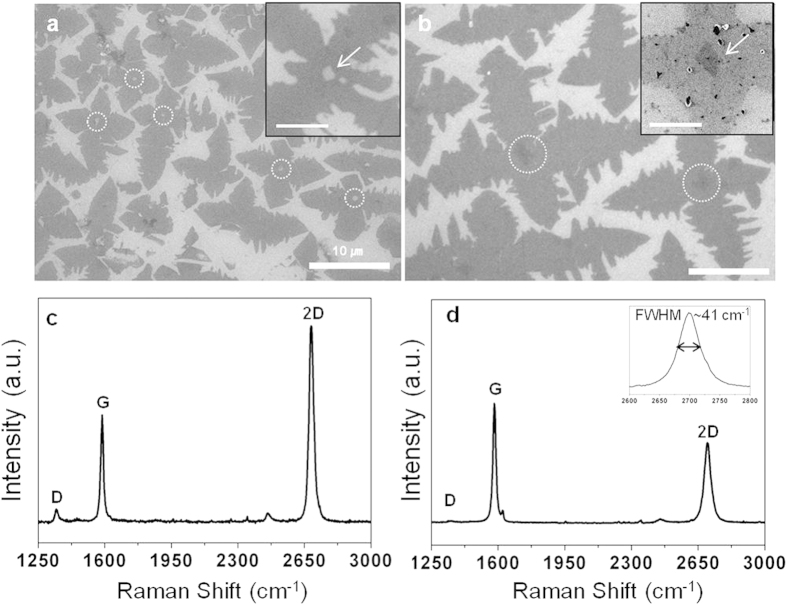
SEM images showing four-lobed graphene domains with (**a**) a hole or (**b**) multilayer graphene in their center. Scale bars represent 10 μm. (Insets) High magnification SEM image of each type of four-lobed graphene. Scale bars represent 2 μm. (**c,d**) Raman spectra taken at the hole and multilayer spot in four-lobed graphene domains, as indicated by the white arrows in insets of (**a,b**), respectively. The inset of (**d**) shows the full width at half maximum (FWHM) of 2D peak, ~41 cm^−1^.

As previously mentioned, the four-lobed graphene domains have a defect in the center, with this thought to be due to a mismatch between the intrinsic six-fold symmetry of graphene and preferential surface diffusion of carbon adatoms on the Cu (001) lattice. That is, the defects produced by this symmetry mismatch provide an activation site for further reaction, with hydrogen in particular having been identified as both an activator for the formation of surface-bound carbon and an etching reagent that affects the shape of the graphene domains^27^. Thus, if the hydrogen plays the role of an etching reagent, then holes are formed at the defects (Indicated by dotted circles in Fig. 5a). On the other hand, if the hydrogen acts as an activator, then additional graphene is formed at defects and ultimately results in multilayered spots (Indicated by dotted circles in Fig. 5b). Consequently, the center of the four-lobed graphene domains can have either a hole or multilayered graphene. The existence of the hole and multilayer spot was corroborated by micro Raman spectroscopy in Fig. 5c,d. The hole in the center of the four-lobed graphene was observed as a high D peak. On the other hand, the ratio of 2D to G peak in Fig. 5d is below 1.0 and the full width at half maximum (FWHM) of 2D peak is ~41 cm^−1^, indicating multilayer graphene^28^. (See the Supplementary Figure S4 for more evidences of hole and multilayer spots in the center of four-lobed graphene domains).

## Conclusions

In summary, we have demonstrated that the crystallographic orientation of Cu substrate differently influences the shape and defects of graphene domains formed on it. Under low pressure, the growth of graphene domains is governed by the underlying Cu substrate, resulting in four- and six-lobed structures on Cu (001) and Cu (102) lattice, respectively. Experimental observations of such structures were corroborated by computational modeling, which identified the preferential diffusion route of carbon adatoms on each copper lattice. In contrast, hexagonal graphene domains were found to form over the entire Cu substrate under atmospheric pressure, suggesting that growth under these conditions is determined by the intrinsic symmetry in the atomic structure of the graphene. Based on these results, the holes and multilayered spots that have frequently observed in the center of four-lobed graphene domains in the past are considered to be due to defects originated from the lattice mismatch between the intrinsic six-fold symmetry of graphene and the preferential surface diffusion of carbon adatoms on Cu (001) lattice. We believe that our research on the surface diffusion directed graphene growth can greatly improve graphene-based engineering by allowing controlled alignment of individual graphene domains, in addition to providing greater insight into producing high-quality large area graphene for use in transparent conducting electrodes, sensors, and nano-electronic devices.

## Methods

### Synthesis of Graphene

Graphene was synthesized by copper-catalyzed chemical vapor deposition under low and atmospheric pressure, using methane as the carbon containing precursor gas. The Cu foil was loaded in the 1 inch quartz tube of tube furnace for annealing process. The tube was evacuated, back filled with hydrogen, and heated to 1000 °C. It was annealed for 1 h. For APCVD, a methane/argon gas mixture (50 ppm methane) of 300 sccm was allowed to flow across the copper at a temperature of 1000 °C, while maintaining 20 sccm hydrogen. The precursor supply time was 15 min during graphene growth. The sample was left to rapidly cool under a hydrogen and argon atmosphere. And, for LPCVD, the temperature was maintained to 1000 °C, and a H_2_ pressure of 40 mTorr was kept with 2 sccm flow. Then, 35 sccm of CH_4_ was introduced in the tube for 10 minutes at a total pressure of 500 mTorr. After exposure to methane, the furnace was cooled to room temperature. The cooling rate was 20 °C/sec.

### Transfer of graphene

After growth, graphene was transferred by a PMMA-assisted wet-transfer method on 300 nm SiO_2_/Si wafer for Raman spectroscopy. A thin layer of PMMA (MicroChem 950 PMMA C3, 3% in chlorobenzene) was spin-coated on an as-synthesized sample at 3000 rpm for 1 min. Since both Cu surfaces were exposed to CH_4_, graphene was grown on both sides of the Cu foil. Excessive PMMA on the back side of PMMA coated graphene was removed by acetone. Subsequently, the sample was placed in a 0.1 M aqueous (NH_4_)_2_SO_4_ to etch off the Cu foil. Generally, the etching process runs overnight. After the Cu foil was completely etched away, graphene with PMMA coating was scooped out from the solution and transferred it into DI water for 10 min to wash away remaining etchants (done three times). Then, SiO_2_/Si wafer was dipped into water and the film was picked up. The PMMA was then removed with acetone and the sample was rinsed several times with DI water.

### Characterizations

Scanning electron microscopy (SEM) images of the synthesized graphene domains were obtained using a field-emission SEM system (JEOL 7600F) operated at acceleration voltages of 10 to 20 kV. Atomic force microscopy (AFM) and electron backscatter diffraction (EBSD) analyses were performed to identify the surface roughness and crystallographic nature of the Cu foil samples, respectively. Raman spectra of the synthesized graphene samples were obtained using a laboratory-made micro-Raman spectroscopy system; the system used the 514.5 nm line of an Ar ion laser as excitation source with a power of ~1 mW. The heating effect of the laser could be neglected at this power level. The laser beam was focused onto the graphene sample using a 50× microscope objective lens (Numerical Aperture = 0.8). The collected scattered light was dispersed using a Shamrock SR 303i spectrometer (1200 grooves/mm) and was detected using a charge-coupled device (CCD) detector.

### Computational modeling

The obtained Cu crystal structures were optimized using density functional theory (DFT) calculations carried out with Dmol package included in materials studio. The calculation of total energy and electronic structure was followed by the geometry optimization using the generalized gradient approximation (GGA) with the Perdew–Burke–Ernzerhof (PBE) functional. The density functional semi-core pseudopotential (DSPP) was employed for the Cu atoms to take relativistic effects into account, while all electrons were considered for the carbon atoms. Further, using linear synchronous transit (LST) and quadratic synchronous transit (QST) methods, a new transition state (TS) search technique for stable configurations of carbon atom is developed, in which the stable configuration is independent on selected initial configurations. Double numerical atomic basis sets augmented with the polarization function (DNP) were used to describe the valence electrons. Two Cu surfaces were simulated using six layer with 54 atoms for (001) and eight layer with 72 atoms for Cu (102), respectively. Each system was sampled with nine (3×3 × 1) in-plane k-points and a vacuum of 10 Å.

## Additional Information

**How to cite this article**: Jung, D. H. *et al.* Surface Diffusion Directed Growth of Anisotropic Graphene Domains on Different Copper Lattices. *Sci. Rep.* **6**, 21136; doi: 10.1038/srep21136 (2016).

## Supplementary Material

Supplementary Information
