# Supplementary material for: Surface Diffusion Directed Growth of Anisotropic Graphene Domains on Different Copper Lattices
Source: Sci Rep. 2016 Feb 17;6:21136. doi: 10.1038/srep21136 (PMC4756662; doi:10.1038/srep21136)
Supplement: Supplementary Information [file srep21136-s1.pdf]

Supplementary Information

Surface Diffusion Directed Growth of Anisotropic Graphene Domains on Different Copper Lattices

Da Hee Jung, Cheong Kang, Ji Eun Nam, Heekyung Jeong, and Jin Seok Lee*

Department of Chemistry, Sookmyung Women's University, Seoul 140-742, Korea

CONTENTS

- **Figure S1.** Size distributions of (a) four-lobed and (b) six-lobed graphene domains, respectively. The average size of four-lobe graphene domains is 11.8 μm, while 8.0 μm for the six-lobed graphene domains. In the case of (102) substrate for the six-lobed graphene, it has six possible ways to diffuse while (001) has four possible ways. Although the calculated energy barrier is smaller for the six-lobed graphene than the four-lobed graphene (Figure 4), which means it relatively facilitates diffusion in the case of (102) substrate, the structural characteristics induces the difference in terms of the domain size. In other words, (102) substrate has more possible ways to diffuse, so that it has slightly smaller size of graphene domains since they are grown from the same amount of carbon source with the six-lobed graphene. Although having the broad distribution of domain size, the four-lobed graphene has slightly larger average size than the six-lobed graphene.

- **Figure S2.** Photo images of the furnace tube after processing at (a) LPCVD and (b) APCVD. Under LPCVD condition, the Cu deposits are clearly observed in the deposition tube because copper has high evaporation rates in vacuum, while there are no Cu deposits in the tube under APCVD condition due to the suppressed sublimation of copper.

- **Figure S3.** Atomic force microscopy (AFM) images of Cu foil after CVD process at (a) low pressure and (b) atmospheric pressure.

- **Figure S4.** Raman maps of (a, b) D/G intensity ratio of four-lobed graphene domains, showing high defects density in the center of the domains, and (c, d) 2D/G intensity ratio, indicating hole and multilayer spots in center area, respectively. Scale bars represent 5 μm.

- **Figure S5.** Raman maps of (a, b) D/G intensity ratio of six-lobed graphene domains, showing uniform defects density over the domains, and (c, d) 2D/G intensity ratio, indicating monolayer over the area. Scale bars represent 5 μm
.

- **Figure S6.** (a) Raman spectrum of general graphene film in our system, and (b) Raman map of D/G intensity, indicating low defects density in our graphene film, which further proves that there is no systematic error in our system.

- **Figure S7.** Computational modeling for the diffusion of carbon adatom on the Cu (111) lattice. Green circles indicate Cu atoms protruding from surface, and gray circle represents carbon adatom adsorbed on Cu lattices. Total electron density plot of Cu (111) lattice. Green circles surrounded by red circles correspond to the surface Cu atoms (region with high electron density), while blue area is region with depleted electron density. The dotted and solid white arrows are the preferential diffusion directions of carbon adatoms with a low energy barrier, while the yellow and black arrows are the poor diffusion directions with a high energy barrier. The energy barriers toward the A (0.30 eV) directions are quite amenable to diffusion; however, the need to pass over a Cu atom protruding from surface, as marked in green, significantly increases the energy barrier in the B (1.30 eV) and C (1.34 eV) directions because of strong electrostatic repulsion. Given this, there are in fact a total six ways in which carbon can diffuse with a relatively low energy barrier.

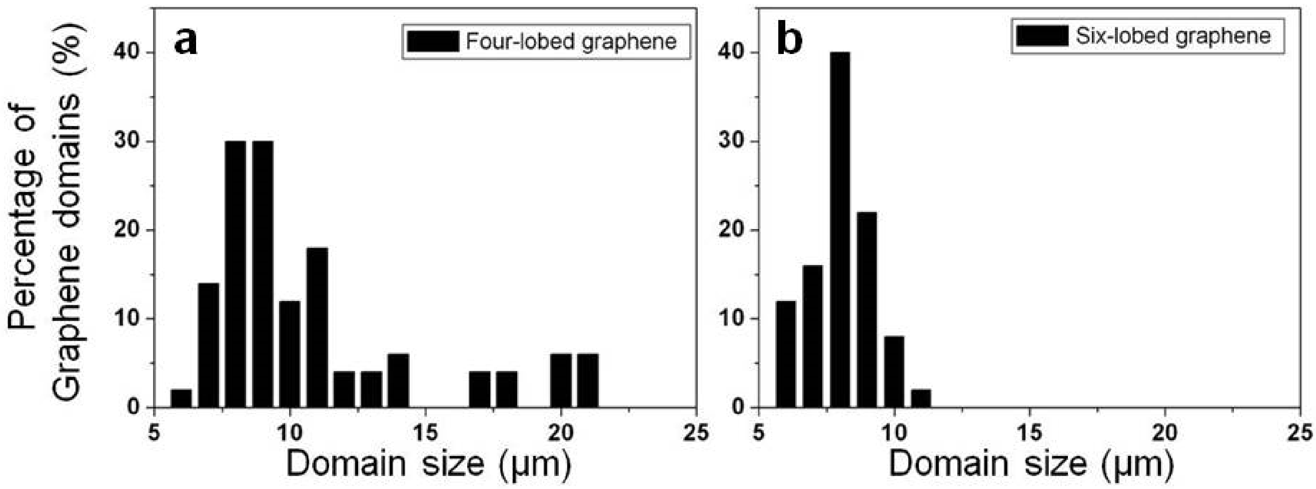

Figure S1. Size distributions of (a) four-lobed and (b) six-lobed graphene domains, respectively. The average size of four-lobe graphene domains is 11.8 μm, while 8.0 μm for the six-lobed graphene domains. In the case of (102) substrate for the six-lobed graphene, it has six possible ways to diffuse while (001) has four possible ways. Although the calculated energy barrier is smaller for the six-lobed graphene than the four-lobed graphene (Figure 4), which means it relatively facilitates diffusion in the case of (102) substrate, the structural characteristics induces the difference in terms of the domain size. In other words, (102) substrate has more possible ways to diffuse, so that it has slightly smaller size of graphene domains since they are grown from the same amount of carbon source with the six-lobed graphene. Although having the broad distribution of domain size, the four-lobed graphene has slightly larger average size than the six-lobed graphene.

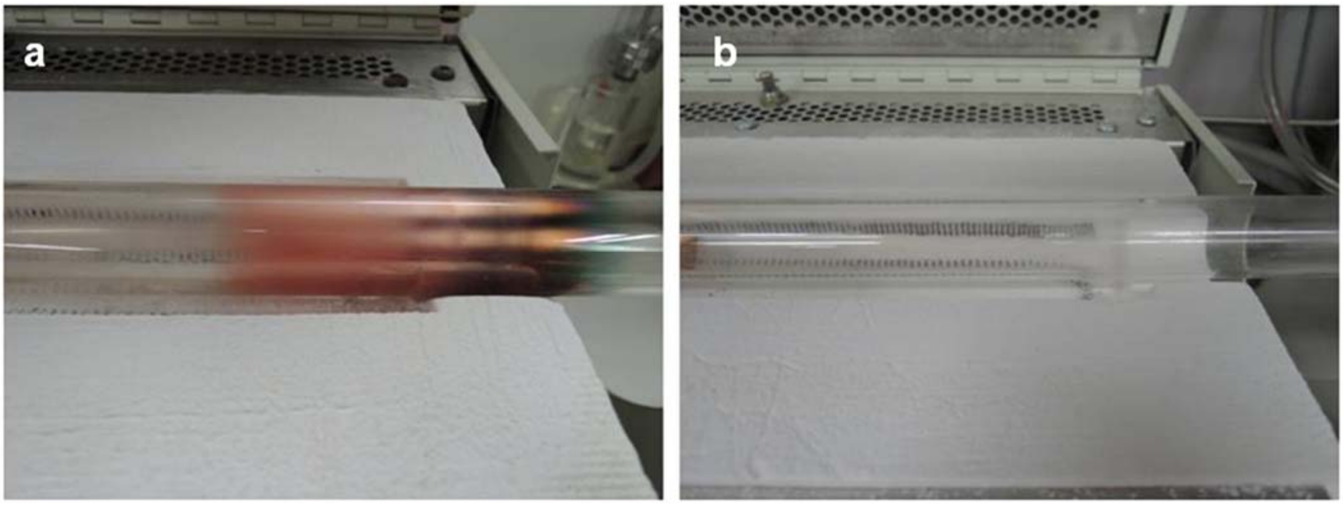

Figure S2. Photo images of the furnace tube after processing at (a) LPCVD and (b) APCVD. Under LPCVD condition, the Cu deposits are clearly observed in the deposition tube because copper has high evaporation rates in vacuum, while there are no Cu deposits in the tube under APCVD condition due to the suppressed sublimation of copper.

In order to identify the effect of background pressure on the surface roughness of the Cu foil, 1 x 1 μm AFM images were obtained. Figure S3a shows an AFM image of the Cu foil processed at low pressure; its root mean square (rms) roughness was relatively high (3.70 nm) due to Cu sublimation at low pressure[1], making the graphene have high defects density over the surface. On the other hand, at atmospheric pressure, the Cu sublimation was suppressed[1], so that the surface roughness decreased to 0.91 nm (Figure S3b). Therefore, the defects density of graphene domains grown at atmospheric pressure is relatively lower than that of graphene grown at low pressure.

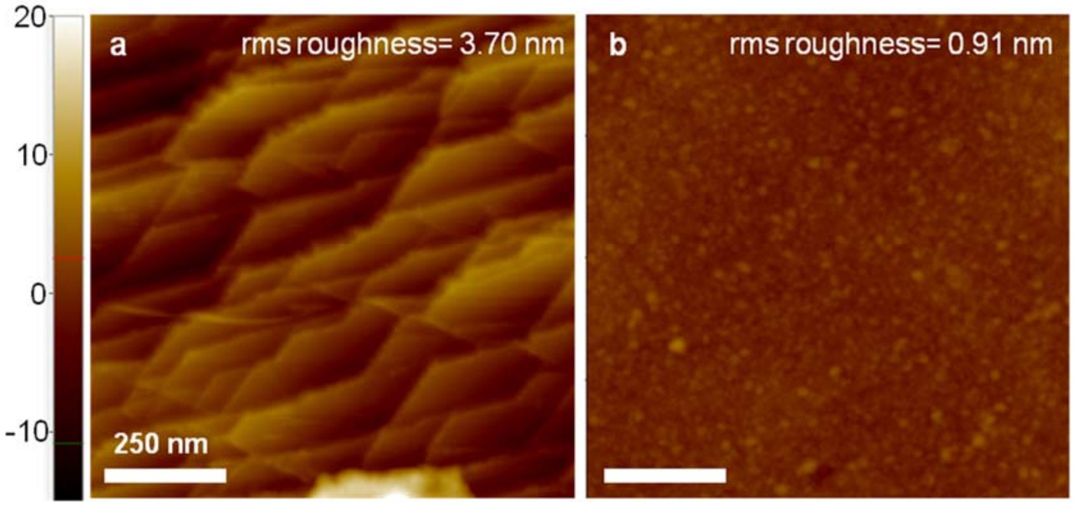

Figure S3. Atomic force microscopy (AFM) images of Cu foil after CVD process at (a) low pressure and (b) atmospheric pressure.

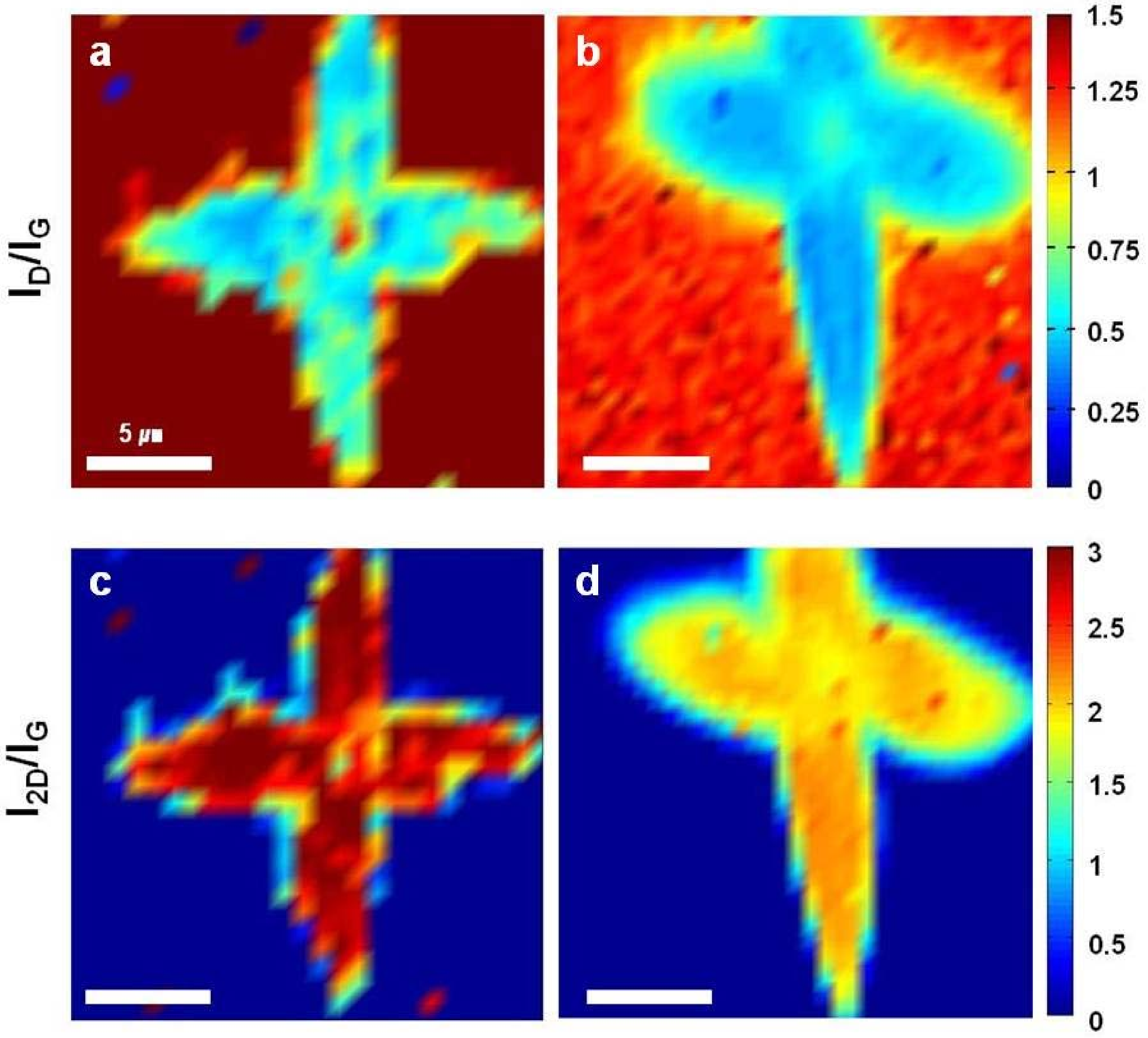

Figure S4. Raman maps of (a, b) D/G intensity ratio of four-lobed graphene domains, showing high defects density in the center of the domains, and (c, d) 2D/G intensity ratio, indicating hole and multilayer spots in center area, respectively. Scale bars represent 5 μm.

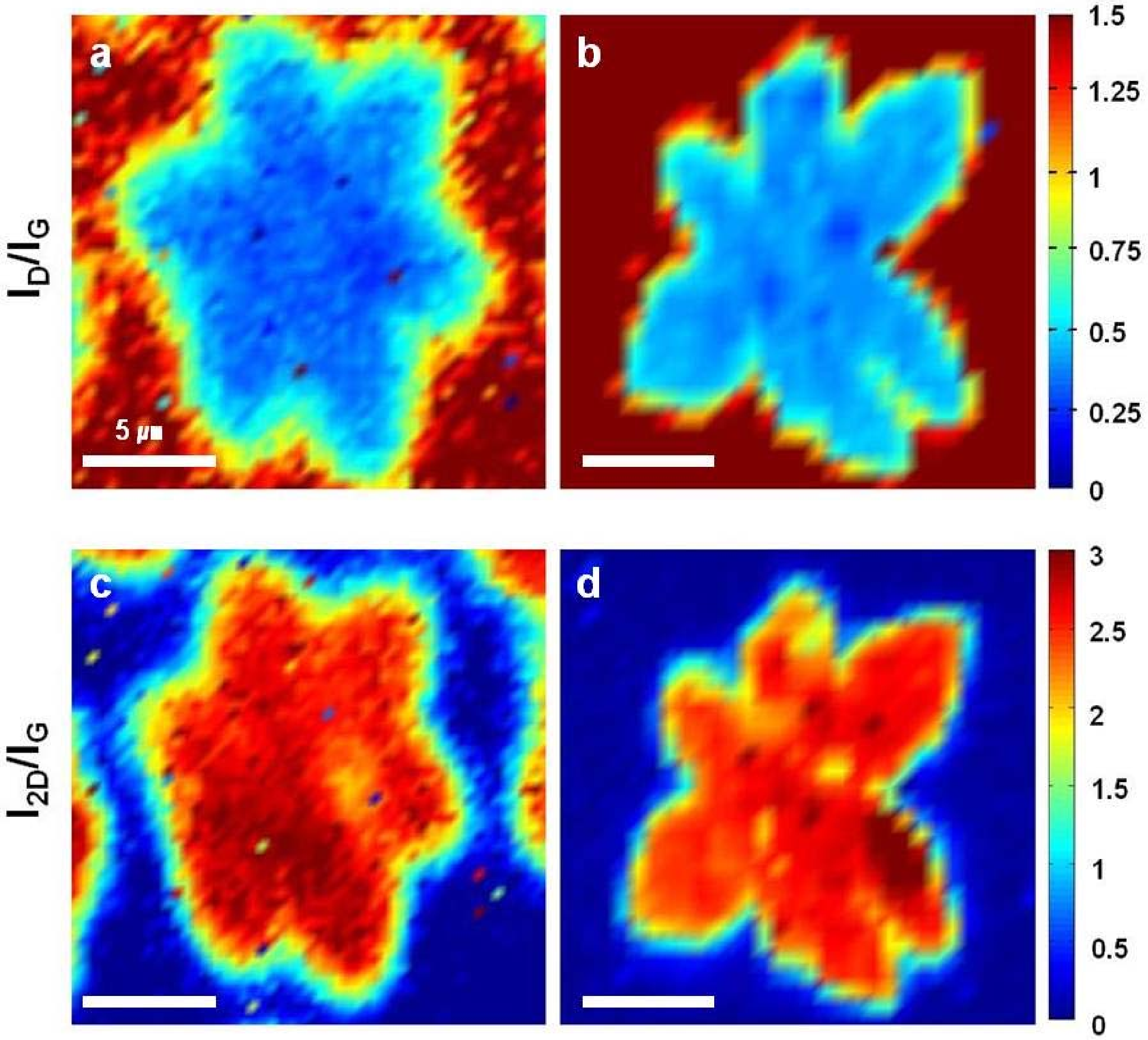

Figure S5. Raman maps of (a, b) D/G intensity ratio of six-lobed graphene domains, showing uniform defects density over the domains, and (c, d) 2D/G intensity ratio, indicating monolayer over the area. Scale bars represent 5 μm.

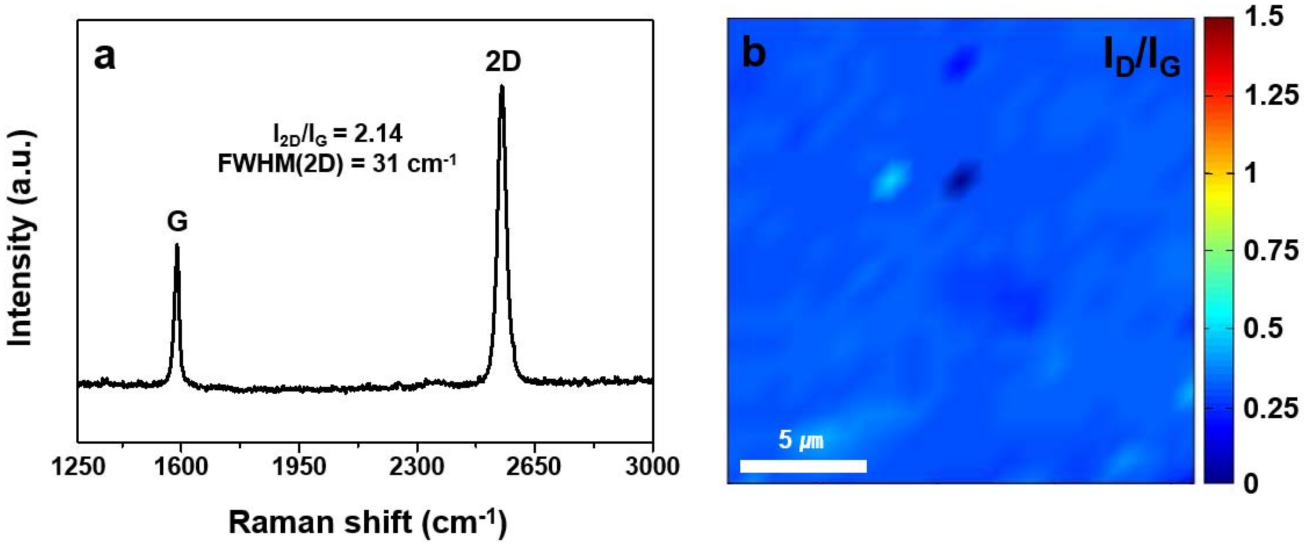

Figure S6. (a) Raman spectrum of general graphene film in our system, and (b) Raman map of D/G intensity, indicating low defects density in our graphene film, which further proves that there is no systematic error in our system.

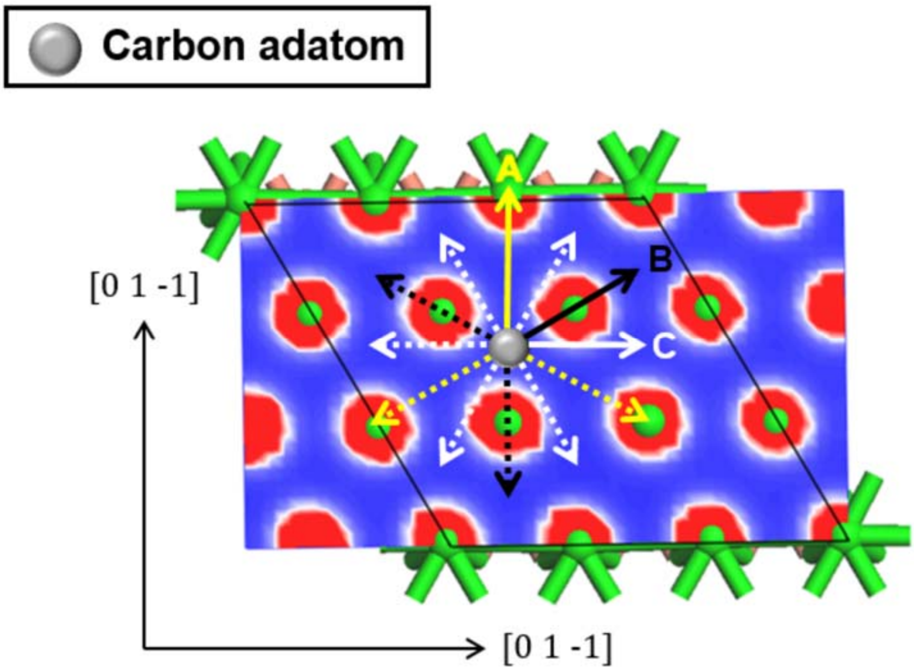

Figure S7. Computational modeling for the diffusion of carbon adatom on the Cu (111) lattice. Green circles indicate Cu atoms protruding from surface, and gray circle represents carbon adatom adsorbed on Cu lattices. Total electron density plot of Cu (111) lattice. Green circles surrounded by red circles correspond to the surface Cu atoms (region with high electron density), while blue area is region with depleted electron density. The dotted and solid white arrows are the preferential diffusion directions of carbon adatoms with a low energy barrier, while the yellow and black arrows are the poor diffusion directions with a high energy barrier. The energy barriers toward the A (0.30 eV) directions are quite amenable to diffusion; however, the need to pass over a Cu atom protruding from surface, as marked in green, significantly increases the energy barrier in the B (1.30 eV) and C (1.34 eV) directions because of strong electrostatic repulsion. Given this, there are in fact a total six ways in which carbon can diffuse with a relatively low energy barrier.

Reference

1. Vlassiouk, I. Fulvio, P. Meyer, H. Lavrik, N. Dai, S. Datskos, P. & Smirnov, S. Large scale atmospheric pressure chemical vapor deposition of graphene. *Carbon* **54**, 58-67 (2013).
